# Microcavity‐Enhanced Polarization Photodetection in Antimony Selenide Nanotube‐Based Near‐Infrared Photodetectors

**DOI:** 10.1002/smsc.202400216

**Published:** 2024-07-12

**Authors:** Songqing Zhang, Khalil As’Ham, Han Wang, Wenwu Pan, Ibrahim Al‐Ani, Huijia Luo, Junliang Liu, Yongling Ren, Haroldo Takashi Hattori, Andrey E. Miroshnichenko, Lorenzo Faraone, Wen Lei

**Affiliations:** ^1^ Department of Electrical, Electronic and Computer Engineering The University of Western Australia 35 Stirling Highway Crawley 6009 Australia; ^2^ School of Engineering and Technology University of New South Wales at Canberra Northcott Drive Campbell ACT 2600 Australia; ^3^ Department of Electronic Engineering School of IOT Engineering Jiangnan University Wuxi 214122 China

**Keywords:** chemical vapor deposition, microcavity resonance effect, near‐infrared polarized photodetectors, polarization photodetection enhancement, Sb_2_Se_3_ nanotubes

## Abstract

This study presents the polarization photodetection enhancement in Sb_2_Se_3_ nanotube (NT)‐based near‐infrared (NIR) photodetectors through simulation‐based and experimental investigations. High‐quality single‐crystal Sb_2_Se_3_ NTs are grown *via* chemical vapor deposition and characterized by using multiple techniques. The optical simulation reveals a remarkable difference in the light absorption ratio (specifically, absorption along the NT/nanowire (NW) against absorption perpendicular to the NT/NW) between Sb_2_Se_3_ NT and NW of the same size in the NIR region. The complementary photodetection experiments present that the fabricated Sb_2_Se_3_ NT photodetector demonstrates enhanced polarization photodetection in the NIR range, as indicated by a significantly increased dichroic ratio (3.03 at 850 nm) compared to that of similar‐sized NW counterpart (1.81 at 850 nm). Additionally, the Sb_2_Se_3_ NT photodetector exhibits exceptional performance, with a high responsivity of 4.18 A W^−1^ and specific detectivity of 8.94 × 10^10^ Jones under 830 nm light illumination. This study provides a comprehensive understanding of the microcavity resonance effect and its role in polarization photodetection enhancement, highlighting the potential of self‐assembled Sb_2_Se_3_ NTs in high‐performance near‐infrared polarized photodetection and other relevant applications.

## Graphical abstract

This study investigates polarization photodetection enhancement in Sb_2_Se_3_ nanotube (NT)‐based near‐infrared (NIR) photodetectors. Through simulation and experimentation, it demonstrates superior polarization performance of Sb_2_Se_3_ NTs over nanowires, reflected in their increased absorption ratio and dichroic ratio in NIR region. It underscores the role of microcavity resonance in enhancing polarization photodetection, offering insights into great potential applications in high‐performance NIR polarized detectors.

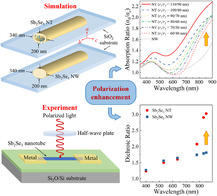

## 1 Introduction

Photodetectors are of utmost importance for a wide range of contemporary applications across civilian, defense, and military sectors.^[^ ^1^, ^2^ ^]^ One remarkable development in the field of photodetection is the integration of polarized capabilities, which appear to be an appealing strategy for enhancing the performance and sensitivity of such devices.^[^ ^3^ ^]^ Polarization‐sensitive photodetectors, which enable precise control over the detection and manipulation of light with specific polarization orientations, has gained increasing significance in biomedical sensing, communication technologies, and advanced imaging systems.^[^ ^4^, ^5^, ^6^ ^]^ Consequently, investigating innovative techniques to enhance the polarization photodetection of sensing systems remains a major field of interest.

Over recent decades, researchers have dedicated efforts to enhance the polarization photodetection of detectors through the utilization of the geometrical anisotropy present in quasi‐1D materials, such as nanowires (NWs) and nanobelts.^[^ ^7^ ^]^ The high aspect ratio morphology and large surface‐to‐volume ratio of these quasi‐1D materials enable optimal light–matter interactions and rapid charge transport along axial directions, resulting in polarization‐dependent optical absorption and generation of photocurrent.^[^ ^8^, ^9^, ^10^ ^]^ Despite these advancements, the polarization performance of photodetectors based on quasi‐1D materials often lags behind that of industrially mature commercial polarization detectors.^[^ ^11^, ^12^ ^]^ Alternatively, some researchers have employed rolled‐up techniques to fabricate tubular microcavities for polarization enhancement. Specifically, the hollow tubular structure forms an optical microcavity that confines incident light along the axial direction.^[^ ^13^ ^]^ This microcavity resonance effect can significantly increase polarization anisotropy in light absorption, for improved photodetector performance.^[^ ^14^ ^]^ However, it is noteworthy that the micrometer‐scale cross sections of these tubular structures may limit the extent of their enhancement of polarization photodetection due to their small aspect ratio (length/diameter).^[^ ^15^, ^16^ ^]^ This small aspect ratio reduces the geometric anisotropy of these structures, thereby leading to lower polarization enhancement.

As a representative quasi‐1D nanostructures, self‐assembled antimony selenide (Sb_2_Se_3_) nanotubes (NTs) provide a promising strategy for enhancing polarization photodetection by synergistically combining the advantages of geometrical anisotropy and the microcavity resonance effect. Additionally, their nanometer‐scale cross sections and intrinsic structural asymmetry give rise to pronounced anisotropic properties, thereby enabling efficient capture and manipulation of polarized light.^[^ ^17^, ^18^ ^]^ These attributes, in conjunction with appropriate bandgap (1.0–1.3 eV) and large light absorption coefficient (> 10^5^ cm^−1^) of Sb_2_Se_3_,^[^ ^19^, ^20^, ^21^, ^22^ ^]^ render it an appealing candidate for the realization of polarization photodetection and enhancement. However, despite their great potential, research on the polarization photodetection enhancement by the microcavity of Sb_2_Se_3_ NTs is notably absent, underscoring the necessity for further research in the realm of microcavity‐enhanced polarization photodetection in self‐assembled NT‐based detectors.

In this study, we present findings on the enhancement of polarization photodetection through the microcavity resonance effect of Sb_2_Se_3_ NTs, as demonstrated by theoretical simulations and photodetection experiments. By utilizing chemical vapor deposition (CVD) technology, we have effectively grown high‐quality monocrystalline Sb_2_Se_3_ NTs. A comprehensive analysis of their hollow tubular structure, crystalline nature, and chemical stoichiometry was conducted using multiple characterization techniques. The optical simulation reveals a significant difference in the light absorption ratio (parallel *vs* perpendicular to Sb_2_Se_3_ NT/NW axis) between Sb_2_Se_3_ NT and NW of identical dimensions, notably in the near‐infrared (NIR) region. This disparity underscores the promising potential of Sb_2_Se_3_ NTs for enhancing polarization photodetection. Complementing these simulation results, the fabricated Sb_2_Se_3_ NT photodetectors demonstrated superior polarized photoresponse, reflected in their considerably higher dichroic ratio (DR) in the NIR region compared to that of similar‐sized Sb_2_Se_3_ NW photodetectors.

## 2 Results and Discussion

**Figure 1:**
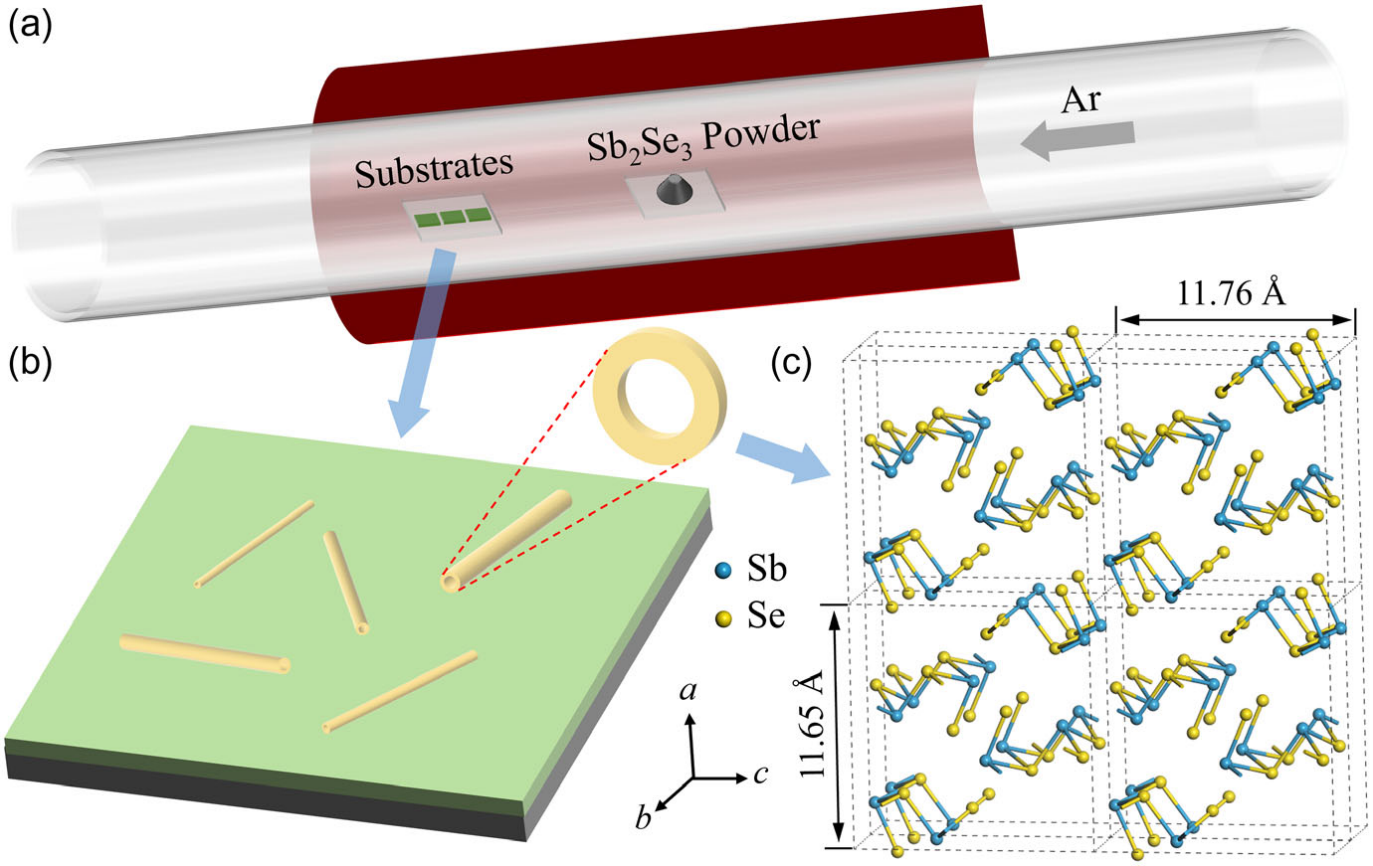
Schematic illustrations of a) CVD setup for Sb_2_Se_3_ NT growth in this study, b) Sb_2_Se_3_ NTs grown on SiO_2_/Si substrate, and c) crystal structure of Sb_2_Se_3_.

The typical CVD setup was utilized to grow Sb_2_Se_3_ NTs in this work, as depicted in **Figure** **1**a. The detailed growth procedure is described in the Methods section, wherein high‐quality Sb_2_Se_3_ NTs were grown under appropriate growth conditions. Figure 1b displays a 3D schematic illustration of Sb_2_Se_3_ NTs grown on the SiO_2_/Si substrate. Figure 1c shows the crystal structure of Sb_2_Se_3_, and the lattice parameters of Sb_2_Se_3_ are *a* = 11.65 Å, *b* = 3.98 Å, and *c* = 11.76 Å. The chain‐like orthorhombic structure along the *b*‐axis in Figure 1c indicates high crystal anisotropic properties of Sb_2_Se_3_, which makes it prone to form 1D nanostructures.

**Figure 2:**
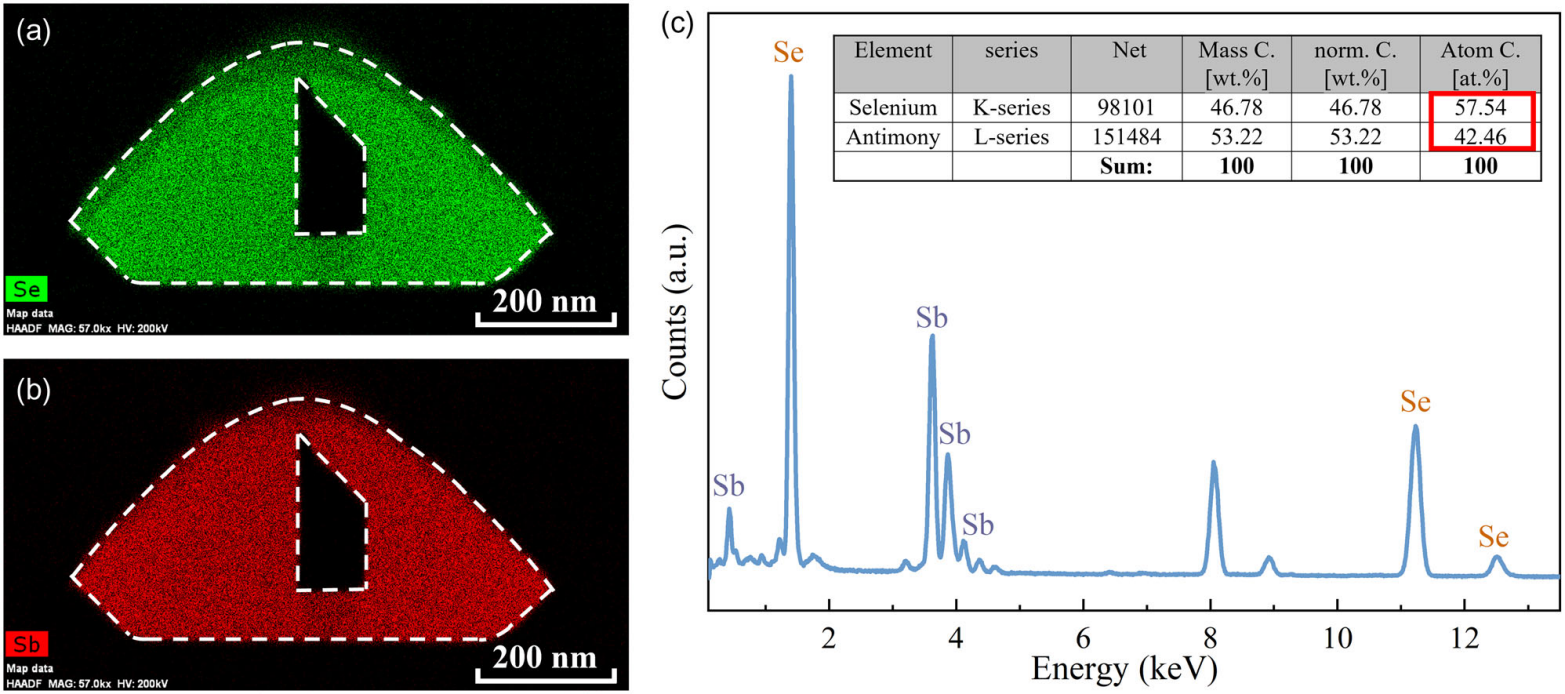
a,b) EDS mapping of a representative self‐assembled Sb_2_Se_3_ NT in the cross‐sectional view and c) its corresponding EDS spectrum.

Scanning electron microscopy (SEM) and transmission electron microscopy (TEM) equipped with selected‐area electron diffraction (SAED)/energy‐dispersive X‐Ray spectroscopy (EDS) were applied in this study to investigate the chemical stoichiometry, hollow tubular shape, and crystal structure of self‐assembled Sb_2_Se_3_ NTs. **Figure** **2**a,b show the EDS mapping of a representative self‐assembled Sb_2_Se_2_ NT in the cross‐sectional view. A hollow microcavity in the Sb_2_Se_3_ NT can be observed, which represents the formation of the self‐assembled tubular structure by CVD growth. In addition, both the distributions of selenium (Se) and antimony (Sb) elements are uniform throughout the cross section of the Sb_2_Se_3_ NT, suggesting high uniformity of the grown NTs. Figure 2c displaces the EDS spectrum derived from the yellow‐bordered rectangular region highlighted in the EDS mapping shown in Figure S1, Supporting Information. Figure S2, Supporting Information, of the SI illustrates the alignment between the observed peaks in Figure 2c and the nominal peaks, in which the spectrum exhibits excellent consistency with the expected peak positions. Quantitative analysis of the EDS spectrum reveals that the chemical composition ratio of Se to Sb in the investigated region is ≈3:2.

**Figure 3:**
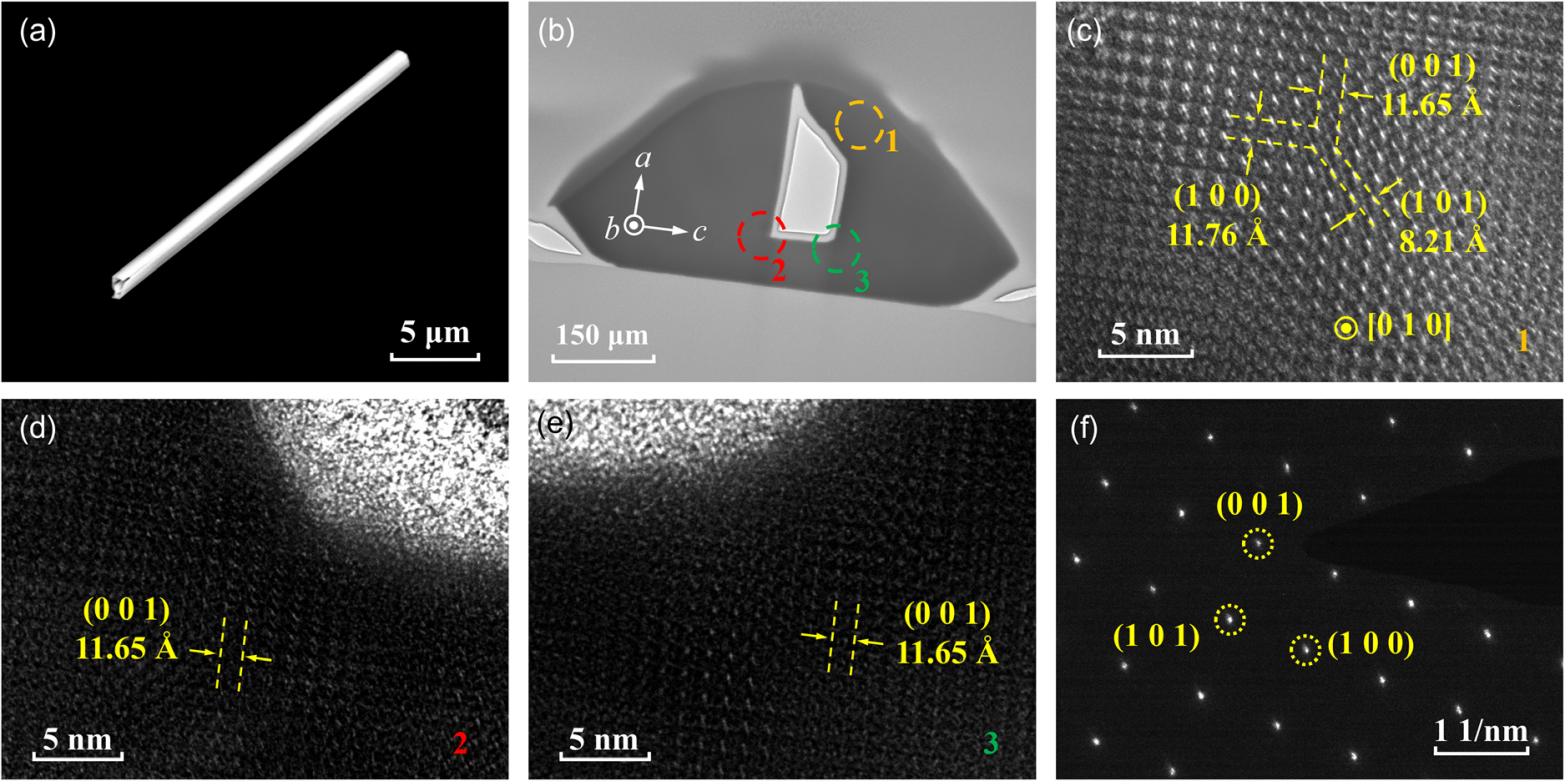
a) SEM image of a representative self‐assembled Sb_2_Se_3_ NT; b) low‐magnification TEM image of the cross‐sectional Sb_2_Se_3_ NT in Figure 2 and c–e) its corresponding HRTEM images obtained at c) circle 1, d) circle 2, and e) circle 3 in (b); and f) SAED pattern obtained at circle 1 in (b).

**Figure** **3**a presents the SEM image of a representative self‐assembled Sb_2_Se_3_ NT, which shows smooth surface and tubular microcavity. Figure 3b exhibits the low‐magnification TEM image of the cross‐sectional Sb_2_Se_3_ NT in Figure 2. Figure 3c–e showcase the high‐resolution TEM (HRTEM) images measured at corresponding regions in Figure 3b. The clear lattice fringes can be observed with the same lattice orientations in all HRTEM images. The interplanar spacings are measured to be 11.76, 11.65, and 8.21 Å, corresponding to the (1 0 0), (0 0 1), and (1 0 1) planes of monocrystalline Sb_2_Se_3_ reported previously, respectively.^[^ ^22^, ^23^, ^24^ ^]^ Hence, the growth orientation of Sb_2_Se_3_ NTs can be identified as [0 1 0], as depicted in Figure 3c. The three lattice directions of the NTs can be determined based on the obtained HRTEM data, as depicted in Figure 3b, which correspond to the three directions illustrated in Figure 1c. Figure 3f depicts the SAED pattern obtained in circle 1 in Figure 3b, and a set of spot patterns can be observed and indexed to the (1 0 0), (0 0 1), and (1 0 1) planes, which are consistent with the orthorhombic phase of Sb_2_Se_3_ in Inorganic Crystal Structure Database (ICSD) Card #30 973. The HRTEM and SAED findings, in conjunction with the preceding EDS analyses, validate both the single‐crystalline nature and accurate chemical stoichiometry of Sb_2_Se_3_ NTs grown *via* the CVD method.

In contrast to forming carbon NTs, which involves the rolling up of layered graphene,^[^ ^25^, ^26^, ^27^ ^]^ Sb_2_Se_3_ NTs are generated through the self‐assembly of Sb_2_Se_3_ chain‐like structures.^[^ ^28^, ^29^ ^]^ This unique formation mechanism results in the uniform lattice orientation throughout various regions within Sb_2_Se_3_ NTs’ cross section, which is confirmed by the same interplanar spacings and lattice directions observed in HRTEM images as depicted in Figure 3c–e. The formation mechanism of the Sb_2_Se_3_ NTs was discussed in Note S1 and Figure S3 and S4, Supporting Information, providing more evidence for the uniformity of lattice orientation and crystallinity in self‐assembled Sb_2_Se_3_ NTs.

We first performed an optical simulation based on the microcavity‐enhanced polarization mechanism to investigate the polarization enhancement enabled by the Sb_2_Se_3_ hollow tubular structure. Subsequently, we fabricated Sb_2_Se_3_ NT/NW photodetectors and conducted polarized photodetection measurements to experimentally validate the simulation results.

**Figure 4:**
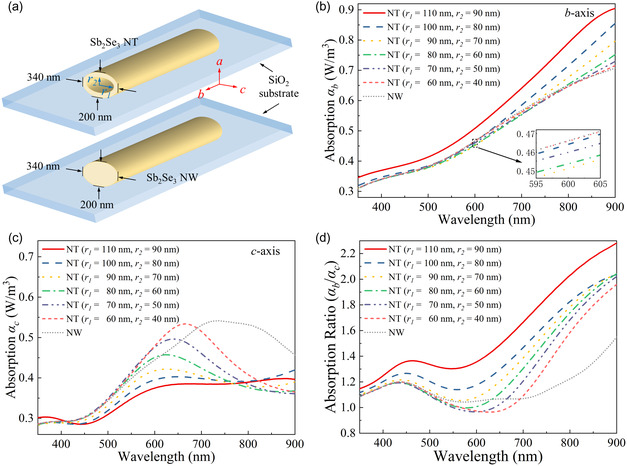
a) Schematic simulation configuration of Sb_2_Se_3_ NT and NW; b,c) simulated absorption spectra of Sb_2_Se_3_ NTs with various microcavity sizes and NW along b) *b*‐axis (*α*_b_) and c) *c*‐axis (*α*_c_); and d) absorption ratio (*α*_b_/*α*_c_) of Sb_2_Se_3_ NTs with various microcavity sizes and NW calculated based on data in (b) and (c).

The finite‐difference time‐domain (FDTD) Lumerical software was employed for the optical simulation to calculate the absorption characteristics of both Sb_2_Se_3_ NT and NW. Because of the uniform lattice orientation within the cross section of Sb_2_Se_3_ NTs, the refractive indexes of Sb_2_Se_3_ along the *b*‐ and *c*‐axis could be utilized for optical simulation, which were derived from density functional theory (DFT) calculations, as shown in Figure S5, Supporting Information. In the simulation, Sb_2_Se_3_ NW/NT was configured to be on a SiO_2_ substrate (analog to the Sb_2_Se_3_ NW/NT grown in this work), as depicted in **Figure** **4**a where the *b*‐axis was found to be parallel to the Sb_2_Se_3_ NTs/NWs, while the *c*‐axis exhibited perpendicularity to the NTs/NWs. It should be noted that the external cross‐sectional dimensions of Sb_2_Se_3_ NT were chosen based on the atomic force microscopy (AFM) and SEM images of the selected Sb_2_Se_3_ NT used for fabricating the polarized photodetector for photodetection measurements later in this work. For accurate comparison, the external cross‐sectional dimensions of the Sb_2_Se_3_ NW were set to be the same as those of the NT, as depicted in Figure 4a. Because of the difficulty in determining the internal tubular shape of the Sb_2_Se_3_ NT (cutting the NT from its cross section would destroy it entirely), we assumed the internal microcavity's shape of the NT to be an ellipse for the sake of efficient simulation and computation. The horizontal radius (*r*_1_) and vertical radius (*r*_2_) of the NT's microcavity were varied to investigate the impact of microcavity size on the NT light absorption spectra. Given that the lengths of Sb_2_Se_3_ NTs/NWs are significantly larger than their cross‐sectional sizes, we assumed an infinite length for NTs/NWs in our simulations. Additionally, the permittivity of SiO_2_ was considered to be 2.1025.^[^ ^30^ ^]^

Figure 4b,c show the simulated absorption spectra of Sb_2_Se_3_ NTs with various microcavity sizes and NW along the *b*‐axis (*α*_b_) and *c*‐axis (*α*_c_). As depicted in Figure 4b, when the magnetic field (H‐field) polarization is along the *b*‐axis, the simulated light absorption of Sb_2_Se_3_ NTs does not exhibit a notable increase compared to that of the NW in the visible light region (*λ* < 700 nm). However, the light absorption of NTs increases significantly as their microcavity size increases and is much larger than that of Sb_2_Se_3_ NW in the NIR region (*λ* > 700 nm). In contrast, when the H‐field polarization is along the *c*‐axis, as shown in Figure 4c, the light absorption of Sb_2_Se_3_ NTs shows a substantial decrease as their microcavity dimensions increase for *λ* < 800 nm, while the light absorption of NTs exhibits a relatively stable behavior, showing negligible variations with changes in their microcavity sizes when *λ* > 800 nm. In addition, the absorption of NTs along the *c*‐axis (with varying microcavity sizes) is generally lower than that of the NW, particularly in the NIR region (*λ* > 700 nm).

For easy comparison, Figure 4d shows the absorption ratio (*α*_b_/*α*_c_) for Sb_2_Se_3_ NTs with various microcavity sizes and NW. Generally, the absorption ratio (*α*_b_/*α*_c_) for Sb_2_Se_3_ NTs is larger than that of Sb_2_Se_3_ NW, especially for NTs with a relatively large microcavity dimension (e.g., *r*_1_ > 90 nm, *r*_2_ > 70 nm). Compared with NWs, this enhanced absorption ratio (*α*_b_/*α*_c_) for Sb_2_Se_3_ NTs can be mainly attributed to the confinement of light within their microcavity along the axial direction^[^ ^13^ ^]^ as a result of their elongated hollow structure and their large aspect ratio. The optical resonance effect is generated through the internal reflection of light along the walls of the NTs, resembling the behavior observed in a Fabry–Pérot cavity.^[^ ^31^, ^32^ ^]^ This resonance is ascribed to the dielectric contrast between the NT material and its ambient environment,^[^ ^16^, ^33^ ^]^ leading to an enhancement of the inner electric field when incident light with an electric field component aligned parallel to the NT axis (*b*‐axis) compared to perpendicularly polarized light (*c*‐axis) as shown in Figure S6, Supporting Information.

More importantly, as shown in Figure 4d, the absorption ratio (*α*_b_/*α*_c_) for Sb_2_Se_3_ NTs increases significantly at longer wavelengths (*λ* > 700 nm) and also increases with increasing the NT microcavity dimensions, leading to a much larger absorption ratio (*α*_b_/*α*_c_) for Sb_2_Se_3_ NTs with a large microcavity size such as *r*_1_ = 110 nm and *r*_2_ = 90 nm and a longer wavelength such as 850 nm. This will lead to a much more sensitive polarization detection in the NIR region for Sb_2_Se_3_ NTs in comparison to Sb_2_Se_3_ NWs, the reason for which will be discussed together with the experimental results later in this work.

**Figure 5:**
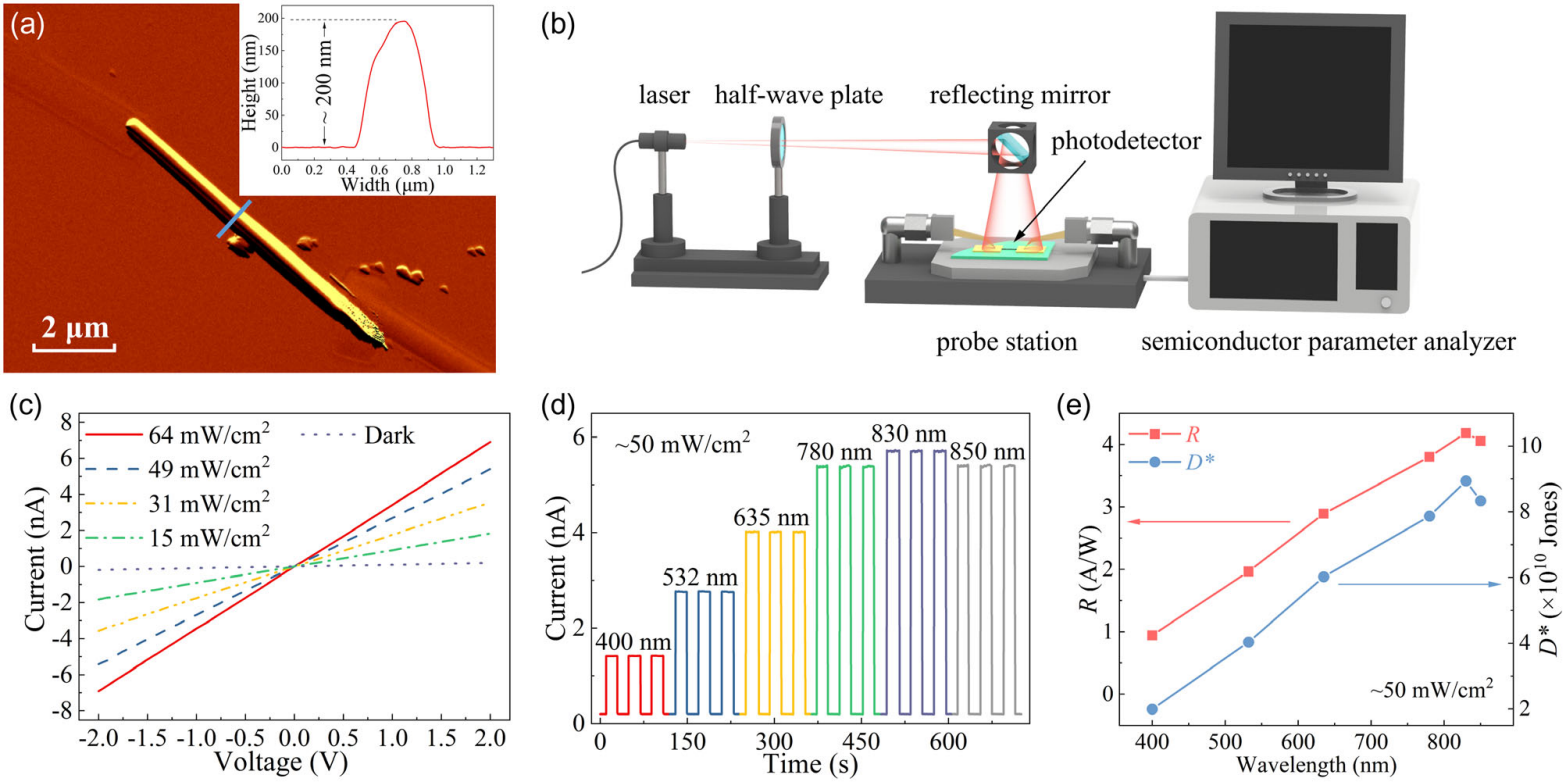
a) AFM image of Sb_2_Se_3_ NT‐PD 1 with the inset displaying its corresponding height profile (along the blue line); b) schematic representation of the photodetection measurement system utilized in this study; c) light intensity‐dependent *I–V*_b_ curves of NT‐PD 1 under 850 nm laser illumination; d) spectral photoswitching behavior of NT‐PD 1 under the laser illumination of different wavelengths (400–850 nm); and e) light wavelength‐dependent responsivity and specific detectivity of NT‐PD 1 calculated based on data in (d). Note: all measurements were implemented at RT; d,e) *V*_b_ = 2 V, *P* = ≈50 mW cm^−2^.

To experimentally validate the polarization enhancement by the microcavity in Sb_2_Se_3_ NTs, several two‐metal‐terminal photoconductive detectors based on both Sb_2_Se_3_ NTs and NWs were fabricated on the SiO_2_/Si substrates. The selected NT, whose morphological sizes were used as the external cross‐sectional dimensions in the preceding optical simulation, was made into photodetectors (marked as NT‐PD 1). **Figure** **5**a presents the AFM image of NT‐PD 1, accompanied by its corresponding height profile along the blue line in the inset. The SEM image of NT‐PD 1 is depicted in Figure S7a, Supporting Information. A corresponding Sb_2_Se_3_ NW photodetector (marked as NW‐PD 1) that has a similar morphological size to NT‐PD 1 was also fabricated to compare the polarization performance disparity between NTs and NWs. Figure S7b,c, Supporting Information, display the SEM and AFM images of Sb_2_Se_3_ NW‐PD 1, accompanied by its corresponding height profile along the blue line in the AFM image.

Additionally, another set of photodetectors utilizing a Sb_2_Se_3_ NT (NT‐PD 2) and a Sb_2_Se_3_ NW (NW‐PD 2) of comparable dimensions were fabricated to validate the consistent enhancement in polarization feature observed in the Sb_2_Se_3_ NT photodetector. Figure S8, Supporting Information, shows the SEM images, AFM images, and their corresponding AFM height profiles of Sb_2_Se_3_ NT‐PD 2 and NW‐PD 2. Based on these SEM and AFM results, the morphological dimensions of NT‐PD 1, NW‐PD 1, NT‐PD 2, and NW‐PD 2 are listed in **Table** **1**.

**Table 1:** Morphological dimensions of NT‐PD 1, NW‐PD 1, NT‐PD 2, and NW‐PD 2.

Set	Device	Height [nm]	Width [nm]	Active length [μm]
Set 1	NT‐PD 1	200	340	6.32
	NW‐PD 1	210	330	6.28
Set 2	NT‐PD 2	270	300	5.61
	NW‐PD 2	270	300	5.83

Figure 5b showcases the schematic representation of the photodetection measurement system. The detailed device measurement setup is described in the Methods section. Regular photodetection measurement of Sb_2_Se_3_ NT‐PD 1 was performed to study the fundamental optoelectronic properties of Sb_2_Se_3_ NTs. It should be mentioned that all testing were carried out at room temperature (RT), applying a bias voltage (*V*_b_) of 2 V, except during the current–bias (*I–V*_b_) measurement.

Figure 5c depicts the *I–V*_b_ characteristics of Sb_2_Se_3_ NT‐PD 1 under the NIR light illumination of 850 nm with different light intensities ranging from 0 to 64 mW cm^−2^. The symmetric and linear nature of the curves signifies the presence of an ohmic‐like contact between the Sb_2_Se_3_ NT and two metal electrodes. The spectral photoswitching characteristics of NT‐PD 1 are illustrated in Figure 5d when illuminated to lasers at a power intensity of ≈50 mW cm^−2^ and varying wavelengths from 400 to 850 nm. The photodetector showcases a wide spectral response encompassing the visible to NIR regions. The reproducible photoresponse suggests the stable and durable behavior of the Sb_2_Se_3_ NT photodetector.

Figure 5e presents the light wavelength‐dependent responsivity (*R*) and specific detectivity (D*) of NT‐PD 1 calculated based on Figure 5d. *R* and D* are crucial optoelectronic figures of merit to evaluate the optical performance of a photodetector, which can be calculated by Equation (1) and (2), respectively. In these equations, *P* represents the light intensity of the incident laser during measurement, *S* refers to the active region of the photodetector, *I*_ph_ is photocurrent that can be obtained by subtracting the dark current (*I*_dark_) from the current with light illumination (*I*_light_), and *q* represents the elementary charge (≈1.6 × 10^−19^ °C).^[^ ^34^ ^]^ From Figure 5e, it is observed that the highest values for *R* and D* are obtained under 830 nm laser illumination with ≈50 mW cm^−2^ light intensity and calculated to be 4.18 A W^−1^ and 8.94 × 10^10^ Jones, respectively

(1) $$R=\frac{I_{\text{ph}}}{PS}$$

(2) $$D^{*}=\frac{I_{\text{ph}}\sqrt{S}}{PS\sqrt{2qI_{\text{dark}}}}=R\sqrt{\frac{S}{2qI_{\text{dark}}}}$$

**Figure 6:**
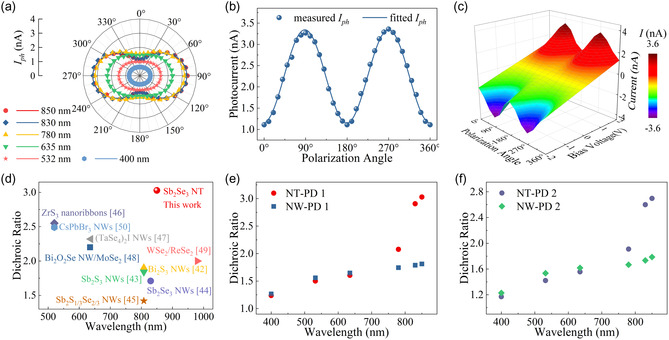
a) Polar plots of polarization‐sensitive photocurrent of NT‐PD 1 under the laser illumination of different wavelengths (400–850 nm); b) polarization angle‐dependent photocurrent under 850 nm laser illumination; c) 3D surface map of the device's measured current under 850 nm laser illumination as a function of voltage bias and polarization angle; d) comparison of device DR values among various low‐dimensional material‐based polarized photodetectors reported; e,f) comparison of device DR values within e) set 1 and f) set 2, respectively. Note: all measurements were implemented at RT; a–c) *V*_b_ = 2 V, *P* = ≈30 mW cm^−2^; data in (a,b) and (e,f) are displayed as the mean ± standard deviation (SD), with the error bars indicating the SD values; the probability (*P*) values are obtained to be less than 0.001 based on one‐way analysis of variance (ANOVA) test with an alpha value of 0.001 and a same size (*n*) of > 10.

To study the polarized properties of Sb_2_Se_3_ NTs, polarized photodetection measurement of NT‐PD 1 was performed. **Figure** **6**a shows the polar plot of polarization‐sensitive photocurrent of NT‐PD 1 measured under the laser illumination of varying wavelengths. The observed elliptical photocurrent curves demonstrate a significant variation in photocurrent as a function of the polarization angle (*θ*). The correlation between *I*_ph_ and *θ* is defined in Equation (3).^[^ ^35^ ^]^ In this equation, *I*_phmax_ represents the maximum *I*_ph_, whereas *I*_phmin_ denotes the minimum *I*_ph_. It should be noted that *I*_phmax_ is achieved when the *θ* of laser light aligns parallel to the Sb_2_Se_3_ NT (*b*‐axis in Figure 3b), whereas *I*_phmin_ occurs when *θ* is perpendicular to the NT (*c*‐axis in Figure 3b)

(3) $$I_{\text{ph}}\left(\theta \right)=I_{\text{phmax}}{\text{cos}}^{2}\left(\theta \right)+I_{\text{phmin}}{\text{sin}}^{2}\left(\theta \right)$$

Figure 6b depicts the polarization angle‐dependent photocurrent of Sb_2_Se_3_ NT‐PD 1 measured under 850 nm laser illumination. Figure 6c presents the 3D surface map of the photodetector's measured current as a function of voltage bias and polarization angle at 850 nm. It is obvious that the sinusoidal wave shapes in Figure 6b and the surface map of Figure 6c are consistent with Equation (3).

Based on Figure 6a, the maximum *R* (*R*_max_), maximum D* (D*_max_), and DR under the laser illumination of varying wavelengths with ≈30 mW cm^−2^ light intensity were calculated to further investigate the spectral response and polarization capability of NT‐PD 1, as depicted in **Table** **2**. Note that DR is a crucial parameter for assessing the polarized properties of a photodetector, which can be calculated by DR = *I*_phmax_/*I*_phmin_. The obtained *R*_max_ and D*_max_ of NT‐PD 1 surpass those of numerous previously reported devices based on low‐dimensional materials.^[^ ^36^, ^37^, ^38^, ^39^, ^40^, ^41^ ^]^ Figure 6d demonstrates the comparison of device DR among various low‐dimensional material‐based polarized photodetectors reported. The calculated DR value (3.03) of Sb_2_Se_3_ NT‐PD 1 at 850 nm is higher than that of numerous polarized photodetectors based on other low‐dimensional materials, including Bi_2_S_3_ NWs (1.90),^[^ ^42^ ^]^ Sb_2_S_3_ NWs (1.84),^[^ ^43^ ^]^ Sb_2_Se_3_ NWs (1.71),^[^ ^44^ ^]^ Sb_2_(S_1/3_Se_2/3_) NWs (1.42),^[^ ^45^ ^]^ ZrS_3_ nanoribbons (2.55),^[^ ^46^ ^]^ (TaSe_4_)_2_I NWs (2.32),^[^ ^47^ ^]^ Bi_2_O_2_Se NW/MoSe_2_ heterostructure (2.2),^[^ ^48^ ^]^ WSe_2_/ReSe_2_ heterostructure (2),^[^ ^49^ ^]^ and CsPbBr_3_ NW (2.49).^[^ ^50^ ^]^ This comparison reveals great potential of CVD‐grown Sb_2_Se_3_ NTs in polarized photodetection.

**Table 2:** Optoelectronic parameters of Sb_2_Se_3_ NT‐PD 1 obtained under the laser illumination of varying wavelengths with ≈30 mW cm^−2^ light intensity.

*λ* [nm]	*P* [mW cm^−2^]	*R*_max_ [A W^−1^]	D*_max_ [Jones]	DR
400	30	0.95	2.01 × 10^10^	1.24
532	28	1.98	4.05 × 10^10^	1.50
635	30	2.91	6.06 × 10^10^	1.60
780	30	3.82	7.91 × 10^10^	2.08
830	29	4.22	9.02 × 10^10^	2.91
850	31	4.15	8.52 × 10^10^	3.03

To verify the enhancement in polarization photodetection by the tubular microcavity of Sb_2_Se_3_ NTs, polarized photodetection measurement of NW‐PD 1, NT‐PD 2, and NW‐PD 2 was also conducted for DR value comparison. Figure S9, Supporting Information, showcases the polar plots of polarization‐sensitive photocurrent of NW‐PD 1, NT‐PD 2, and NW‐PD 2. The observed photocurrent curves exhibit consistency with Equation (3). Figure 6e,f depict the comparison of device DR values within set 1 and set 2, respectively. Evidently, the Sb_2_Se_3_ NT photodetectors (NT‐PD 1 and NT‐PD 2) demonstrate higher DR values in the NIR range compared to those of Sb_2_Se_3_ NW photodetectors (NW‐PD 1 and NW‐PD 2). NT‐PD 1 demonstrates the highest polarization enhancement at 850 nm, with a DR value reaching 3.03, as compared to that of 1.81 for NW‐PD 1. Similarly, the DR value of NT‐PD 2 reaches 2.70 at 850 nm, in contrast to that of 1.79 for NW‐PD 2.

As shown in Figure 6e,f, it can be observed that photodetectors based on both Sb_2_Se_3_ NTs (NT‐PD 1 and NT‐PD 2) and NWs (NW‐PD 1 and NW‐PD 2) demonstrate comparable DR values at *λ* < 635 nm (visible region). This phenomenon may be attributed to both NT and NW structures that behave as magnetic dipoles, with weaker electric dipole responses in the visible region compared to those in the NIR region, akin to dielectric spherical particles as referenced in previous studies.^[^ ^51^, ^52^, ^53^ ^]^ In such short wavelength range, the electric field of NTs/NWs is negligible, regardless of whether the incident light is parallel to (*b*‐axis) or perpendicular to (*c*‐axis) the NTs/NWs. As the absorption is proportional to the electric field, the similarity in the small electric fields of NTs and NWs in Figure S6, Supporting Information, indicates that the absorptions of the NT along the *b*‐ and *c*‐axis are comparable to those of the NW, respectively, resulting in a similar absorption ratio and, consequently, similar DR values for the Sb_2_Se_3_ NT and NW. However, the NT photodetectors exhibit higher DR values compared to those of the NW photodetectors at *λ* > 635 nm (NIR region), which correlates well with the much larger absorption ratio (*α*_b_/*α*_c_) for Sb_2_Se_3_ NTs in the NIR region as shown in Figure 4d. To provide a visual representation, Figure S10, Supporting Information, illustrates the comparison between the experimental device DR values for set 1 and the preceding simulated absorption ratios (*r*_1_ = 90 nm, *r*_2_ = 70 nm). In the NIR region, the hollow tubular microcavity in NTs reduces the electric field along the *c*‐axis compared to that of NWs due to volumetric field enhancement.^[^ ^54^ ^]^ The effects on the electric field in NTs are more pronounced in the NIR region because NTs exhibit lower volumetric field enhancement along the *c*‐axis as shown in Figure S6, Supporting Information, resulting in reduced absorption along the *c*‐axis. This increases the absorption ratio (*α*_b_/*α*_c_) and DR values in the NIR region.

The calculated DR values of Sb_2_Se_3_ NT‐PD 1 and the simulated absorption ratio of Sb_2_Se_3_ NTs demonstrate a similar enhancement in the NIR range compared to those of Sb_2_Se_3_ NW, as depicted in Figure S10, Supporting Information. This enhancement, both experimentally and theoretically, confirms that the polarization property of photodetectors is enhanced by the hollow tubular structure. That is, the distinctive hollow microcavity in NTs enables an increase of polarization anisotropy, thereby enhancing the polarization photodetection in our Sb_2_Se_3_ NT photodetector.

## 3 Conclusion and Outlook

In conclusion, our study has demonstrated the significant influence of the microcavity resonance effect of Sb_2_Se_3_ NTs on the enhancement of polarization photodetection in optical sensing applications. By utilizing the CVD technology, we achieved the growth of high‐quality self‐assembly Sb_2_Se_3_ NTs with single crystal and correct chemical stoichiometry, enabling a comprehensive investigation of their distinctive structural and optical characteristics. The optical simulation demonstrated the substantial difference in the light absorption ratio between Sb_2_Se_3_ NT and NW with comparable sizes, especially in the NIR range, revealing great potential of Sb_2_Se_3_ NT in polarization enhancement. These simulation results were supported by complementary photodetection measurements of the fabricated Sb_2_Se_3_ NT photodetectors, which showed the significantly increased DR value in the NIR region in comparison to their NW counterparts, which underscores the notable polarization enhancement capabilities of Sb_2_Se_3_ NTs. In addition, the NT photodetector exhibited exceptional performance, demonstrated by its high responsivity of 4.18 A W^−1^ and specific detectivity of 8.94 × 10^10^ Jones under 830 nm laser illumination at RT. This study contributes to the advancement of knowledge regarding the relationship between the microcavity resonance effect and the polarization photodetection enhancement of Sb_2_Se_3_ NTs, paving the way for their promising applications in high‐performance polarized photodetection and related technologies. It is anticipated that a similar polarization enhancement can be attained in Bi_2_S_3_ and Sb_2_S_3_ NT‐based photodetectors, given their striking similarity in crystal structure to Sb_2_Se_3_. This microcavity‐enhanced polarization effect may potentially manifest in other anisotropic and isotropic materials, warranting more investigation in subsequent studies and thus laying the foundation for the potential industrial application in polarized photodetection of these tubular structures.

## 4 Experimental Section

4.1

4.1.1

##### CVD Growth of Sb_2_Se_3_ NTs/NWs

CVD growth of self‐assembly Sb_2_Se_3_ NTs/NWs was implemented using a 16‐inch horizontal furnace (Linderberg/Blue M), a 1‐inch‐diameter quartz tube, and an Ar carrier gas flow system. The standard CVD procedure is as follows: 5 mg of 99.99% pure Sb_2_Se_3_ powder and multiple SiO_2_/Si substrates (5 × 5 mm^2^) were positioned at the furnace center and 12–15 cm downstream from the furnace center, respectively. After sealing and vacuuming the quartz tube with a vacuum pump, the tube was flushed multiple times with ultrahigh‐purity Ar carrier gas in order to eliminate oxygen and moisture contaminants. Following that, the furnace was preheated to 500 °C at a heating rate of 25 °C min^−1^ in the absence of Ar gas flow. It was then maintained at this temperature for varying growth time from 30 to 60 min with a growth pressure of 3.65 Torr and an Ar carrier flow rate of 150 sccm. Afterward, the furnace cooled to RT naturally without Ar gas flow.

##### Material Characterization of Sb_2_Se_3_ NTs

SEM (FEI Verios 460 L) and AFM (Witec alpha 300RA^+^) were utilized to identify the morphologies of the Sb_2_Se_3_ NTs grown in this work. TEM (FEI Titan G2 80–200 TEM/STEM) integrated with SAED/EDS were employed to study the chemical stoichiometry and crystal structure of the grown Sb_2_Se_3_ NTs. Notably, the EDS spectrum and calculated chemical stoichiometry were produced by QUANTAX EDS software (BRUKER ESPRIT 1.9). The preparation of the TEM specimens for the Sb_2_Se_3_ NTs involved the application of focused ion beam (FIB, FEI Helios NanoLab G3 CX) techniques.

##### Optical Simulation

Absorption calculations were conducted using the Lumerical FDTD solution. The refractive indexes of Sb_2_Se_3_ along different directions for the simulations were obtained by our previous DFT calculations,^[^ ^44^ ^]^ while the permittivity of SiO_2_ was considered to be 2.1025.^[^ ^30^ ^]^ The simulation region was 2 × 2 × 2 μm^3^, while the mesh volume was 5 × 5 × 10 μm^3^. A plane wave source ranging from 350 to 900 nm was utilized to calculate the absorption and electric field distribution of the simulated structure. The absorption at different polarizations was calculated using absorption box placed around the simulated NT/NW.

##### Photodetection Experiments

FIB equipment was employed to fabricate the two platinum electrodes (100 × 100 μm) of the Sb_2_Se_3_ NT‐ and NW‐based photodetectors. A probe station (Lakeshore TTPX) equipped with a semiconductor parameter analyzer (Keithley 4200) was employed to apply the bias voltage and collect the measurement data of the Sb_2_Se_3_ NT and NW photodetectors. Note that the probes in the probe station are made of beryllium–copper alloy. Six linearly polarized lasers spanning emission wavelengths from 400 to 850 nm were utilized to assess the optoelectronic capabilities of the Sb_2_Se_3_ NT/NW photodetectors. A half‐wave plate was applied to alter the polarization angle of the incident laser light during the polarized measurement. During non‐polarized measurements, the half‐wave plate was also employed to align the polarized orientation of all lasers parallel to the Sb_2_Se_3_ NT/NW devices, mitigating the influence of diverse polarizations among the light sources.

##### Statistical Analysis

Data in Figure 6a,b,e,f, and Figure S9 and S10, Supporting Information, were presented as mean ± SD (error bars), calculated with MATLAB R2021a. One‐way ANOVA test was performed using Excel 365 to assess statistical significance between groups, with *n* > 10 and alpha = 0.001. The analysis yielded *P* values < 0.001, revealing statistically significant differences between groups. Note that data in Figure S10, Supporting Information, were normalized prior to analysis.

## Conflict of Interest

The authors declare no conflict of interest.

## Supporting information

Supplementary Material

## Data Availability

The data that support the findings of this study are available from the corresponding author upon reasonable request.
