# Supplementary material for: Microcavity‐Enhanced Polarization Photodetection in Antimony Selenide Nanotube‐Based Near‐Infrared Photodetectors
Source: Small Sci. 2024 Jul 12;4(10):2400216. doi: 10.1002/smsc.202400216 (PMC11935067; doi:10.1002/smsc.202400216)
Supplement: Supplementary file 1 — Supplementary Material [file SMSC-4-2400216-s001.pdf]

# Supplementary Information

## Microcavity-enhanced polarization photodetection in antimony selenide nanotube-based near-infrared photodetectors

Songqing Zhang[1], Khalil As'Ham[2], Han Wang[1], Wenwu Pan[1], Ibrahim Al-Ani[2], Huijia Luo[1], Junliang Liu[1,3], Yongling Ren[1], Haroldo Takashi Hattori[2], Andrey E. Miroshnichenko[2], Lorenzo Faraone[1], Wen Lei[1*]

*1. Department of Electrical, Electronic and Computer Engineering, The University of Western Australia, 35 Stirling Highway, Crawley 6009, Australia*

*2. School of Engineering and Technology, University of New South Wales at Canberra, Northcott Drive, Campbell, ACT 2600, Australia*

*3. Department of Electronic Engineering, School of IOT engineering, Jiangnan University, Wuxi 214122, China*

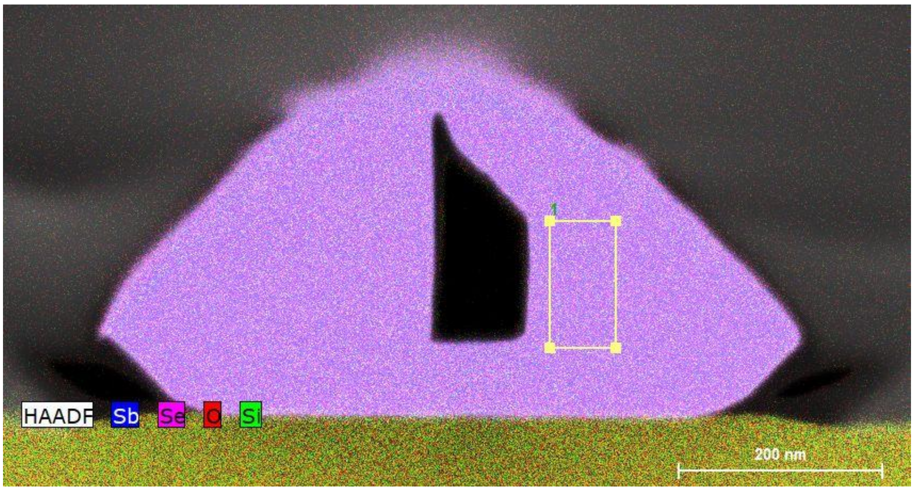


**Figure S1.** High-angle annular dark-field imaging (HAADF) image in conjunction with EDS mapping of Sb, Se, O, and Si elements.

* Electronic mail: wen.lei@uwa.edu.au

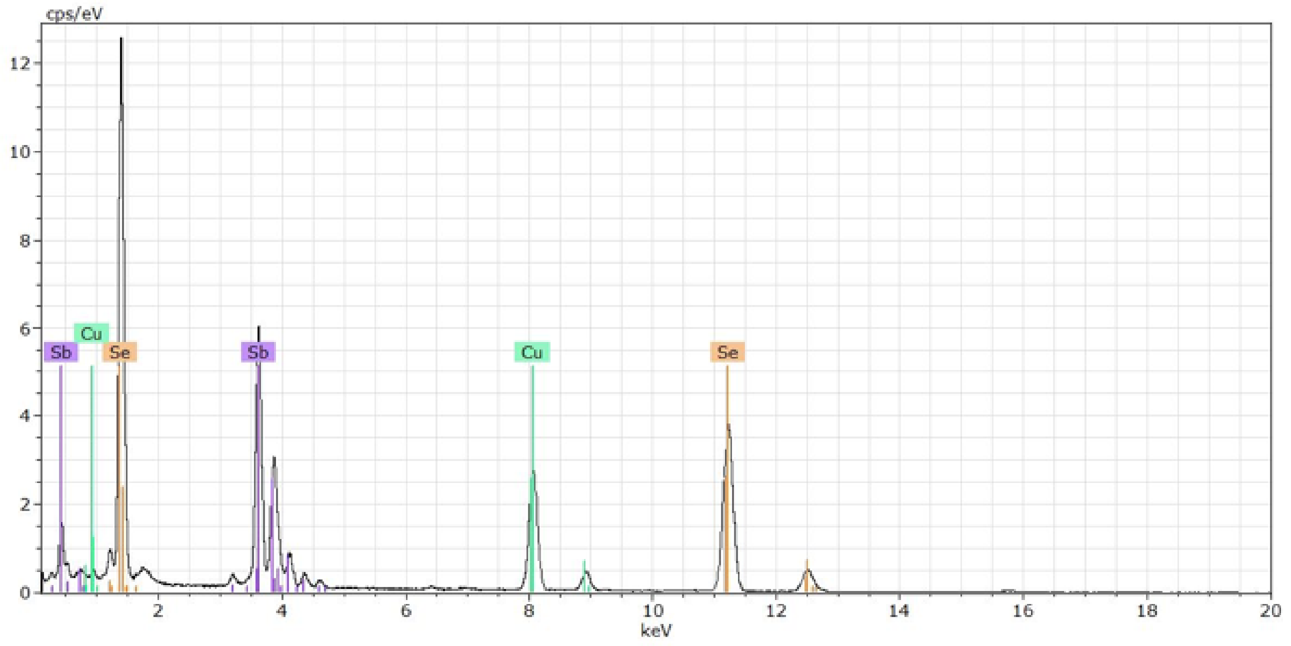


**Figure S2.** Alignment between the experimental EDS peaks and the nominal peaks.

**Note S1**. Formation mechanism of $Sb_2Se_3$ nanotubes

To validate the formation mechanism of self-assembled $Sb_2Se_3$ nanotubes (NTs), several growth experiments were conducted under various growth conditions to analyze the fundamental chemical vapor deposition (CVD) growth mechanism of $Sb_2Se_3$ NTs. Scanning electron microscopy (SEM) was used to analyze the morphological evolution of the CVD-grown $Sb_2Se_3$ nanostructures. Figure S3(a)-(d) demonstrate the top-view SEM images of $Sb_2Se_3$ nanostructures grown with a growth time of 30 minutes, 40 minutes, 50 minutes, and 60 minutes, respectively. It should be noted that other growth parameters were kept constant to ensure precise comparison, as detailed in the Methods. These SEM images provide significant insights into the evolution of $Sb_2Se_3$ NT formation.

During CVD growth, vaporized $Sb_2Se_3$ molecules are generated by the evaporation of $Sb_2Se_3$ powder through furnace heating, then brought downstream to deposit and decompose on the substrate surface by the argon (Ar) carrier gas flow. The decomposed Sb and Se atoms then nucleate in appropriated sites and eventually form $Sb_2Se_3$ nanostructures. At a short growth time of 30 minutes, deposited $Sb_2Se_3$ atoms/molecules first grew into nanowires (NWs) or nanorods (NRs) due to the chain-like crystal structure of $Sb_2Se_3$, as depicted in Figure S3(a). Simultaneously, a considerable number of vaporized $Sb_2Se_3$ atoms/molecules nucleated in the vicinity of the outer surface of the NWs/NRs. This phenomenon might be attributed to the enhanced dissipation of latent heat in these regions during the solidification process.[1] When the growth time was extended to 40 minutes, multiple clusters of

NWs/NRs were formed to enclose the rough hollow tubular structures, as shown in Figure S3(b). Upon extending the growth time to 50 minutes, the adjacent NWs/NRs progressively merged together by the continual deposition of vaporized $Sb_2Se_3$ atoms/molecules into the grooves between NW/NR clusters. The rough tubular structures became smoother, as depicted in Figure S3(c). By increasing the growth time to 60 minutes, the well-grown $Sb_2Se_3$ NTs were formed with smooth surfaces and distinct central microcavities (Figure S3(d)).

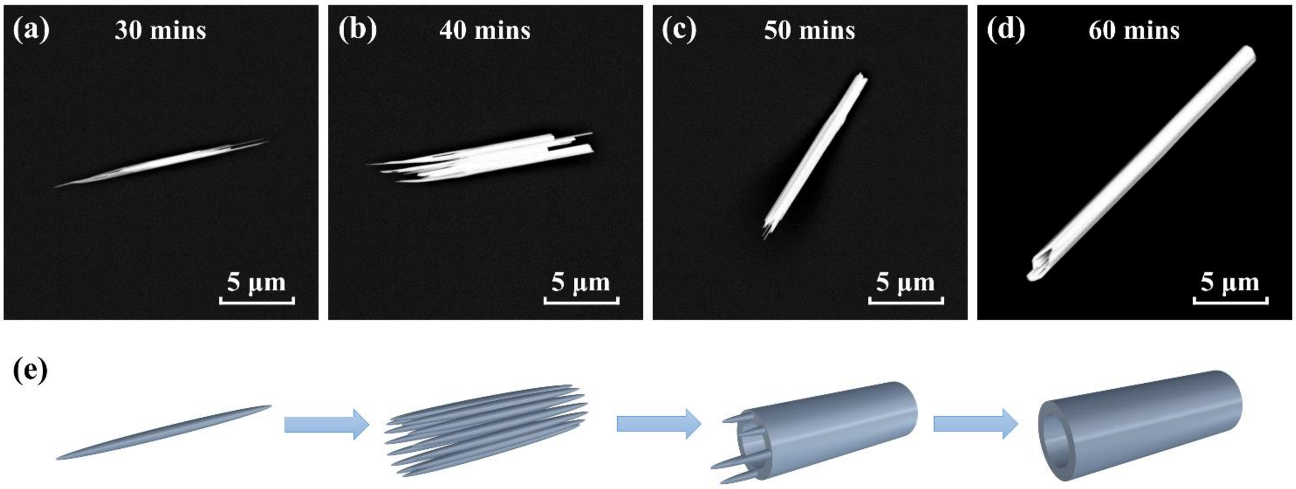


**Figure S3.** Top-view SEM images of $Sb_2Se_3$ nanostructures grown on $SiO_2$/Si substrate with a growth time of (a) 30 minutes, (b) 40 minutes, (c) 50 minutes, and (d) 60 minutes, respectively; (e) Schematic diagram of proposed growth procedure model for $Sb_2Se_3$ NT growth.

A proposed growth procedure model was introduced to study the growth mechanism and dynamics of $Sb_2Se_3$ NTs based on the above SEM images,[2] as depicted in Figure S3(e). A series of SEM images were acquired from a top-side perspective by tilting the Sb2Se3 samples to verify this growth model, as shown in Figure S4 below. It can be observed that various $Sb_2Se_3$ tubular structures possess clear microcavities and extended NW/NR tips. These tubular structures are in the under-developed or transitional states to form $Sb_2Se_3$ NTs, which precisely match the states depicted in Figure S3(b)-(c). These results offer additional proof in favor of the proposed growth mechanism for the growth of $Sb_2Se_3$ NTs and confirm the above formation mechanism.

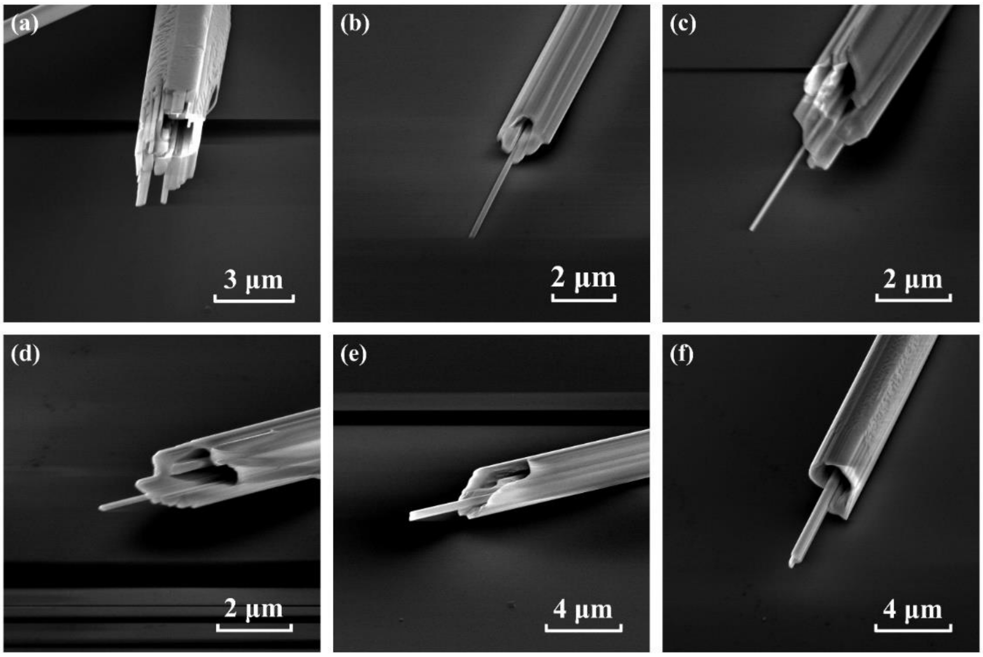


**Figure S4.** SEM images of representative under-developed $Sb_2Se_3$ NTs from a top-side view by tilting the $Sb_2Se_3$ samples (52°).

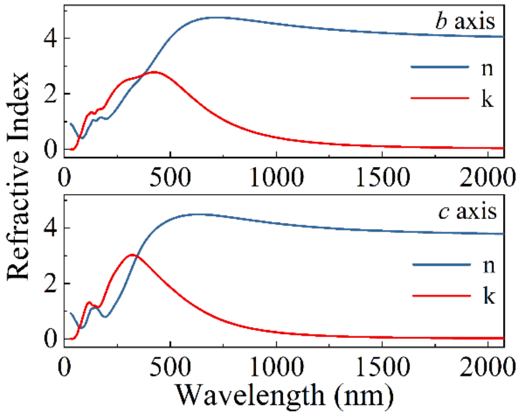


**Figure S5.** Refractive indexes of bulk $Sb_2Se_3$ along *b*-axis and *c*-axis calculated by DFT calculation.

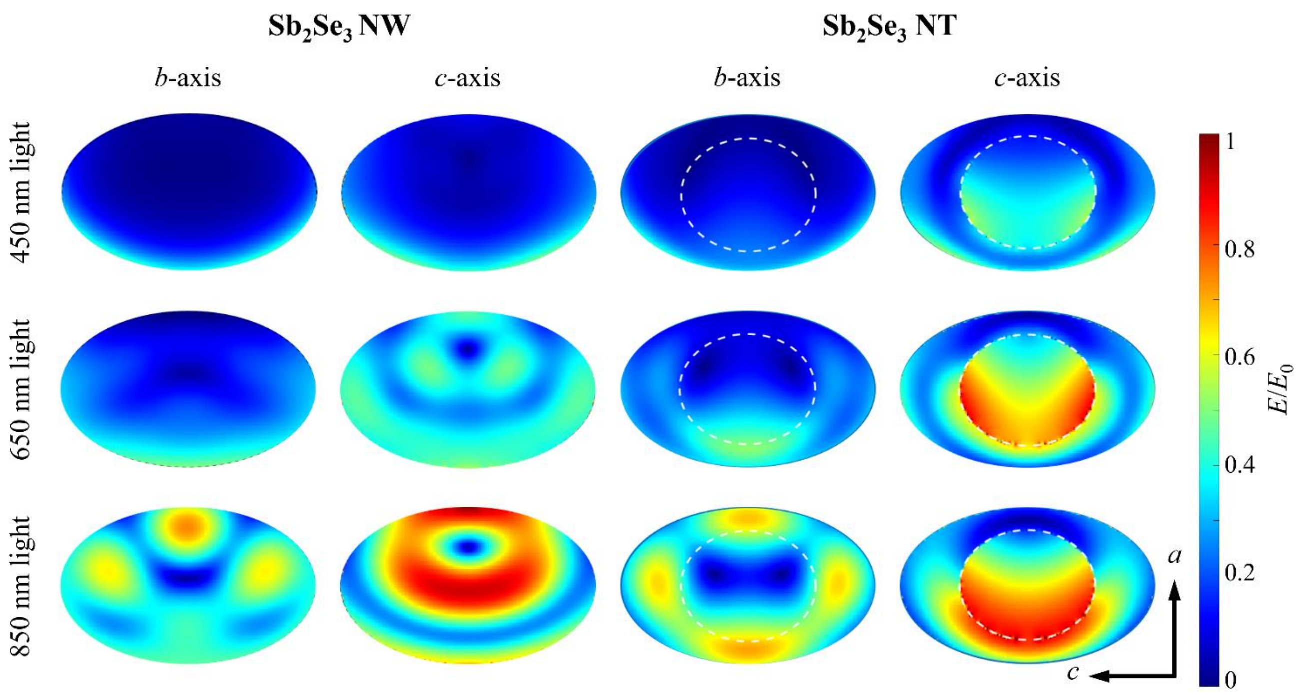


**Figure S6.** Electric field distribution of $Sb_2Se_3$ NW and NT ($r_1$ = 90 nm, $r_2$ = 70 nm) along *b*-axis and *c*-axis.

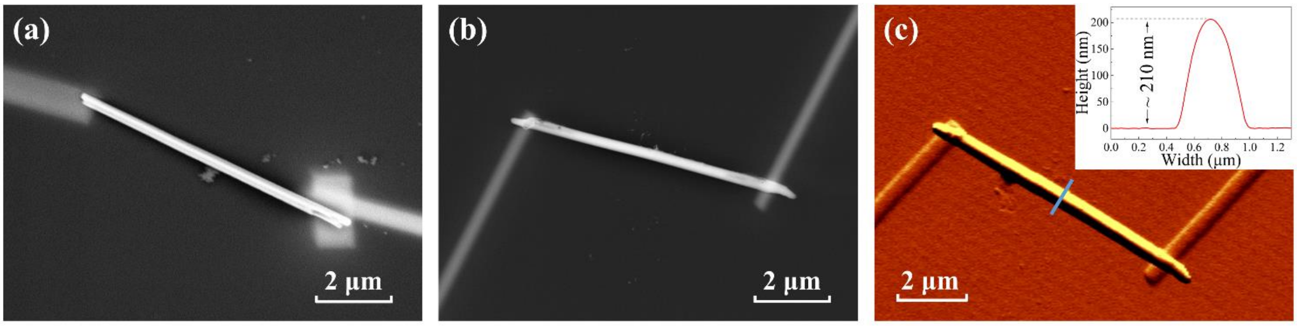


**Figure S7.** (a) SEM image of $Sb_2Se_3$ NT-PD 1; (b) SEM and (c) AFM images of $Sb_2Se_3$ NW-PD 1 (the inset is its corresponding height profile along the blue line in (c)).

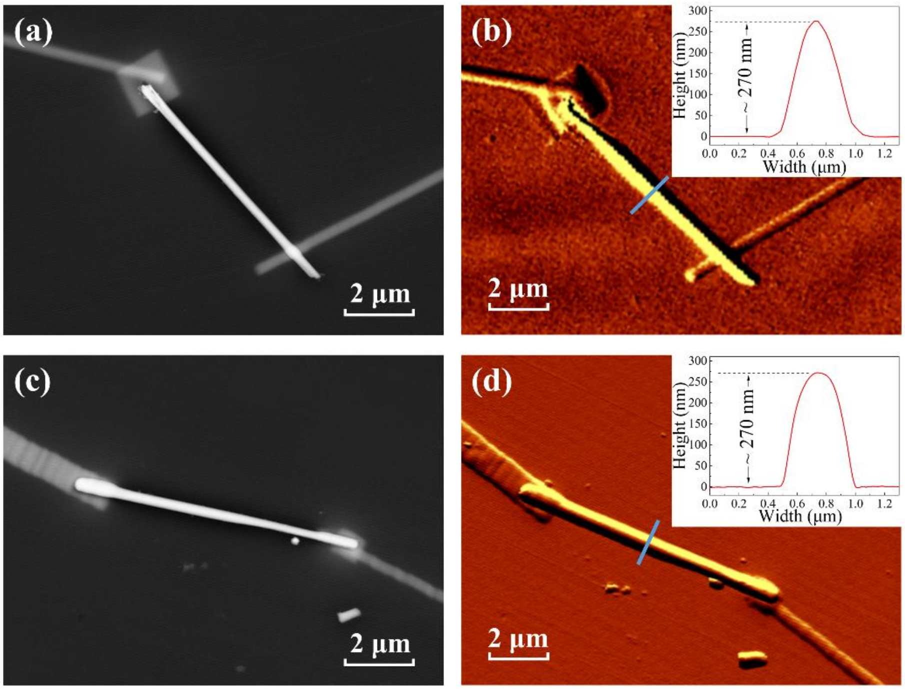


**Figure S8.** (a) SEM and (b) AFM images of $Sb_2Se_3$ NT-PD 2 (the inset is its corresponding height profile along the blue line in (b)); (c) SEM and (d) AFM images of $Sb_2Se_3$ NW-PD 2 (the inset is its corresponding height profile along the blue line in (d)).

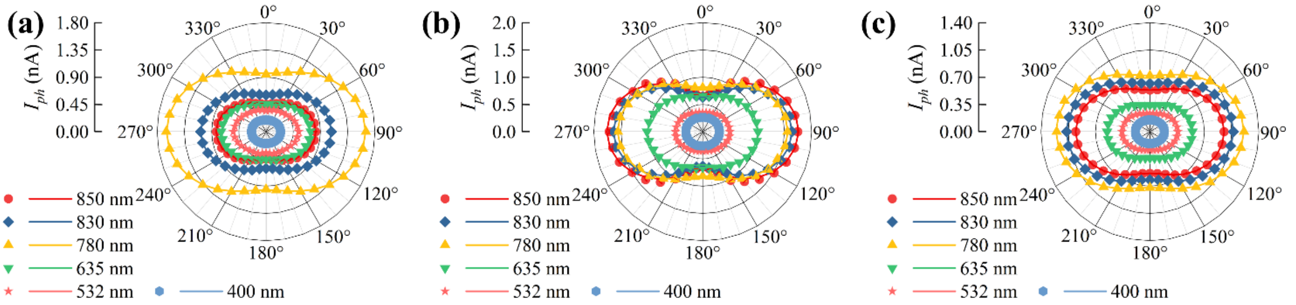


**Figure S9.** Polar plots of polarization-sensitive photocurrent of (a) NW-PD 1, (b) NT-PD 2, and (c) NW-PD 2, respectively; Note: all measurements were implemented at RT with a $V_b$ of 2 V and a light intensity of ~ 30 mW cm$^{-2}$; data in (a)-(c) are displayed as the mean ± SD (error bars); the P values are obtained to be less than 0.001 based on one-way ANOVA test with alpha = 0.001 and n > 10.

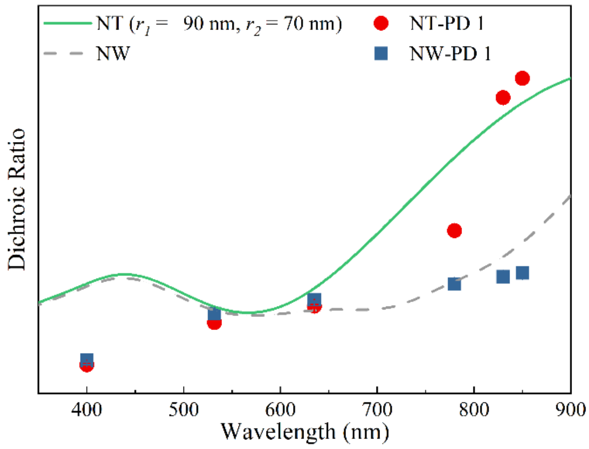


**Figure S10.** Comparison of normalized device DR values of set 1 with the simulated normalized absorption ratio of $Sb_2Se_3$ NW and NT ($r_1$ = 90 nm, $r_2$ = 70 nm). Note: DR values are displayed as the mean ± SD (error bars); the P values are obtained to be less than 0.001 based on one-way ANOVA test with alpha = 0.001 and n > 10.

# Reference


1. Attolini, G., C. Paorici, and P. Ramasamy, *Skeletal and hollow crystals of cadmium sulphide grown under time-increasing supersaturation.* Journal of crystal growth, 1986. **78**(1): p. 181-184.
2. Yang, J., et al., *A chain-structure nanotube: Growth and characterization of single-crystal Sb2S3 nanotubes via a chemical vapor transport reaction.* Advanced Materials, 2004. **16**(8): p. 713-716.
